# Supplementary material for: Analysis of chimeric reads characterises the diverse targetome of AGO2-mediated regulation
Source: Sci Rep. 2023 Dec 21;13:22895. doi: 10.1038/s41598-023-49757-z (PMC10739727; doi:10.1038/s41598-023-49757-z)
Supplement: Supplementary file 3 — Supplementary Information. [file 41598_2023_49757_MOESM3_ESM.docx]

**Analysis of chimeric reads characterises the diverse targetome of AGO2-mediated regulation.**

**Suppl. Table ST1: List of AGO2-CLASH (separate file)**

**Suppl. Table ST2: List of AGO2-eCLIP (separate file)**

**Human cell culture**

The Human Embryonic Kidney 293 T-Rex FlpIn (HEK293T) with inducible expression of hAGO2-PTH was gratefully provided by Tollervey lab [1]. Cells were cultured in DMEM supplemented with 10% FBS in an atmosphere of 5% CO2, 37°C.

**The CLASH protocol**

Three replicate experiments were performed from Flp-In™ T-REx™-HEK293 hAGO2-PTH, each one grown to 40% confluency at 4 x 15 cm plates and hAGO2-PTH expression was induced with 0.5 μg/ml Doxycycline for 36 hours. Three replicate experiments were done by using WT Flp-In™ T-REx™-HEK293 as background control. The UV crosslinking was performed at 254 nm, 2 x 200 mJ/cm^2^ on ice in a Stratalinker with a brief interval between each round UV exposure. Each replicate was harvested into 10 ml lysis buffer (50mM Tris-HCl (pH7.8), 300mM NaCl, 1% NP40 (v/v), 5mM EDTA (pH 8.0), and 10% glycerol (v/v), 5mM BME) with protease inhibitors (cOmplete™ Protease Inhibitor Cocktail) and incubated on ice for 15 minutes. The insoluble fraction was removed by centrifugation at 6400 x g for 30 minutes at 4°C. The supernatant was flash frozen and stored at -80°C.  For each replicate experiment we used 20mg of M-270 Epoxy beads (ThermoFisher Scientific) coated with 8µg of rabbit IgG (Sigma-Aldrich-I5006) per mg of beads as described in [2]. The beads were washed 3x times with 5 ml of lysis buffer. Washed beads were mixed and incubated with freshly thawed UV crosslinked cell lysates for 1 hour at 4°C with gentle rotation. The beads were sedimented by using a magnetic stand and supernatant was removed. The beads with bound proteins were washed 1x with 10 ml of wash buffer (50mM Tris-HCl (pH7.8), 300mM NaCl, 0.5% NP40 (v/v), 5mM MgCl2, and 2.5% glycerol (v/v), 5mM BME), 1 x with 10ml of wash buffer 2 (50mM Tris-HCl (pH7.8), 800mM NaCl, 0.5% NP40 (v/v), 10mM MgCl2, and 2.5% glycerol (v/v), 5mM BME), and 3 consecutive washes 10 mins at 4°C with 10 ml of PNK buffer (50mM Tris-HCl (pH7.8), 50mM NaCl, 0.5% NP40 (v/v), 10mM MgCl2, and 5mM BME). Each time, the beads were gently resuspended and sedimented on a magnetic stand. Beads were then incubated in 1ml of PNK buffer containing 1U of Rnase A+T1 mix (Agilent- 400720) for 5 min at 20°C with mild shaking, the supernatant containing the RNase mix was discarded by sedimenting the beads. The protein RNA complexes were eluted by resuspending the beads in 500 μl NiNTA wash buffer (Ni-WB1, 50mM Tris-HCl (pH7.8), 300mM NaCl, 0.1% NP40 (v/v), 10mM Imidazole (pH 8.0), 6M guanidine hydrochloride, 5mM BME) and incubated for 10 min at RT. The elution was repeated two more times with 0.5 ml and 1 ml of Ni-WB1. All elutions were pooled and mixed with 100 μl of Ni-NTA bead slurry pre-equilibrated in Ni-WB1 and incubated for 2 hours at 4°C with rotation. High stringency gravity flow washes were carried out in a snap cap columns sequentially with 3x with 750μl Ni-WBI, 3x with 750μl Ni-WB2 (Ni-WB1 without 6M guanidine hydrochloride) and 3x with 750μl volumes of PNK buffer. To add phosphate groups at 5’ termini of crosslinked RNAs the mixtures were incubated with 2U of PNK in 80μl reaction volume for 2.5 hrs at 20°C and subsequently washed sequentially 3x with 750μl Ni-WB1, 3x with 750μl Ni-WB2 and 3x with 750μl of PNK. Intermolecular RNA ligations were carried out with 2U of T4 RNA ligase I mix (NEB) overnight at 16°C while shaking at 1000 RPM. Beads were washed thoroughly and incubated with 2 U of Alkaline phosphatase FastAP (ThermoFisher Scientific) in 80 ul reaction volume for 45 mins at 20°C. The 3’ end linker miRCat-33 **(Supplementary Table ST3)** was ligated in the presence of 40 U T4 RNA ligase 2, truncated, K227Q (NEB) overnight with intermittent shaking at 1000 RPM at 16°C. Beads were washed 3x with 750μl of Ni-WB1, 3x with 750μl of Ni-WB2 and 3x with 750μl of PNK buffer by transferring the beads to a new microfuge tube after each wash to remove residual non-ligated linkers. After the last wash, beads were resuspended in 1 ml of PNK buffer. 100μl of the resuspended beads in PNK buffer beads was used for the detection of crosslinked RNAs by radiolabeling using 2U T4 PNK, 3μl 32P-Gamma-ATP, RNase inhibitor (20U) in a reaction volume of 80μl. The radiolabeled samples were resolved on a gradient precast gel and transferred to a nitrocellulose membrane (Biorad). The signals were detected by autoradiography by using an X-ray film (Amersham hyperfilm™). **(Supplementary Figure S1)** The rest of the beads were sedimented and mixed 200μl with elution buffer (Ni-EB; 50mM Tris-HCl (pH7.8), 50mM NaCl, 0.1% NP40 (v/v), 150mM imidazole (pH 8.0), and 5mM BME) to release protein-RNA complexes and incubated at RT for 5 min with mild shaking. The elution was repeated twice with 200μl and 600μl Ni-EB. The eluates were pooled and transferred to a new tube and the proteins were TCA precipitated by adding 2μg BSA, 200μl of TCA (100%) and incubation on ice for 60 min. The protein RNA complex was pelleted by spinning the samples at 21000 g for 10 min at 4°C. The pellets were resuspended in 20μl ddH2O, and treated with 8U of proteinase-K (NEB) for 1 hour at 37°C, in a final reaction volume of 100μl. The reaction was supplemented with 100μl of Urea PK buffer (50mM Tris-HCl pH 7.8, 50mM NaCl, 0.1% NP-40, 1% SDS, 5mM EDTA, 7M Urea) and incubated for an additional hour at 37°C. RNA was purified by Phenol-Chloroform-Isoamyl Alcohol (PCI) and precipitated with equal volumes of isopropanol overnight at -20°C, sedimented by centrifugation at 21000g for 30 mins at 4°C, followed by two washes with 1 ml 75% ice cold ethanol. The RNA pellets were resuspended in 15 μl phosphorylation mixture (T4 RNA ligase buffer, 1mM ATP,  2U T4 PNK) and incubated at 37°C for 30 mins. The samples were supplemented with 5’ adapter ligation mix (T4 RNA ligase buffer, 1mM ATP, 5μM 5’ barcoded adapter **(Supplementary Table ST3)**, 1U RNA ligase I (NEB)) and incubated overnight at 16°C. Linker ligated RNA was purified by PCI and precipitated with equal volumes of 100% Isopropanol at -20°C overnight. The RNA pellet was washed twice with 75% ice cold ethanol and resuspended in 13 μl of RT mix (0.5 mM dNTPs, 0.5μM miRCat-33 RT primer). Samples were incubated at 70°C for 3 min and snap chilled on ice for 5 mins, 7 μl of RT-mixII was added to the reaction (First strand buffer-1X, 5mM DTT (0.1M), 2U RNasein, 200U Superscript RTIII) and incubated at 50°C for 60 mins. The RT samples were treated with RNase H (5U, ThermoFisher Scientific) for 30 mins at 37°C.

The library was amplified by PCR using P3-P5 primer pairs **(Supplementary Table ST3)**, with Taq polymerase (TaKaRa LA) for 21 cycles. The libraries were size selected using 3% MetaPhor agarose and Qiagen gel purification kit using manufacturer’s protocol. Downstream quality checks were carried out using Qubit (ThermoFisher Scientific) and Bioanalyzer (Agilent Technologies). The library was analyzed by high-throughput sequencing on Illumina Next seq 500.


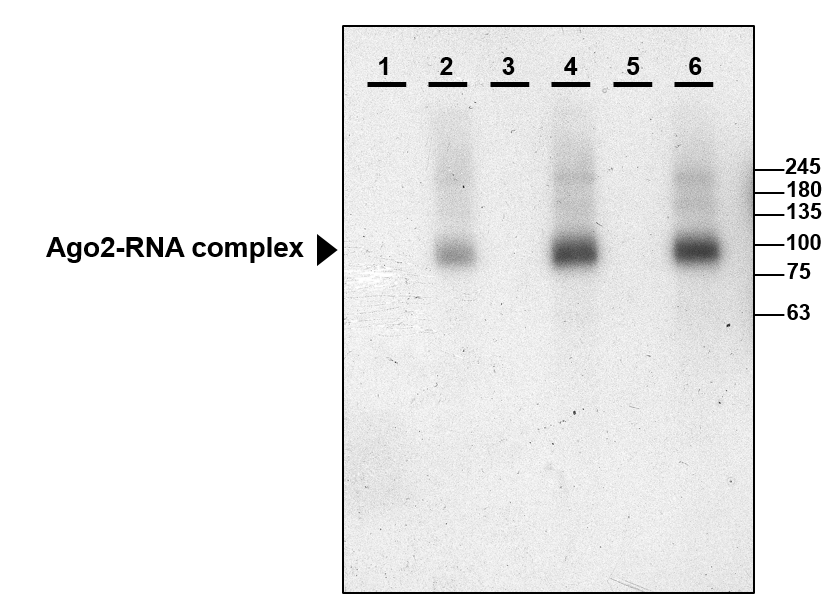


**Supplementary Figure S1:** Autoradiography of 32P-labeled RNA–protein complexes resolved by SDS-PAGE (4–20% gradient gel). Arrow indicates the expected PTH-Ago2–RNA. 10% of the beads were used for imaging purposes, while the rest of the RBP-RNA complex were directly treated with Proteinase K on the beads.

Lanes 1, 3 and 5: No antibody control

Lanes 2, 4 and 6: PTH-Ago2 IP

*A full gel image is available as Supplementary Figure S8*

**QuantSeq analysis of the effect of selected miRNA downregulation on mRNA steady state levels**

Flp-In™ T-REx™-HEK293 cells were seeded at a concentration of 0.5 x 10^5^ cells per well in a 24 well plate. At ~40% confluency the cells were transfected with anti-miR oligonucleotides (IDT) at final concentration 30 nM (**Supplementary Table ST3**) using 1.5 μl of Lipofectamine RNAiMAX (ThermoFischer scientific) per well. As a control we used cells transfected with the nontargeting RNA (NC1). After 24 hrs of incubation at 37°C, cells were lysed with 500 μl TriZol and RNAs were isolated according to the manufacturer’s instructions. The downregulation of target miRNAs was monitored by TaqMan MicroRNA Assays according to manufacturer protocol (Thermo Fisher Scientific) and normalised to endogenous control RNU44 (**Supplementary Figure S3**). 10 ng of the isolated total RNA was used for cDNA preparation using Applied Biosystem™, cDNA Reverse Transcription Kit with RNase inhibitor according to manufacturer instructions.

**Reporter assays for validation of small RNA function by using renilla-luciferase reporter**

To examine the efficiency of guide-target binding in Flp-In™ T-REx™-HEK293 Cell lines, we constructed the target reporter genes with the psi-CHECK-2 vector (Promega). The 50bp long predicted seed sequences were commercially synthesized (Sigma) and inserted between the XhoI and NotI restriction sites within the 3’ untranslated region (UTR) of the *Renilla luciferase* gene. Cells were seeded onto a 24-well plate at density of 1 X 10^5^ cells per well and transfected with 500 ng of the psi-CHECK-2 reporter construct together with final concentration 100 nM of the guide duplex (miRNA mimic) using Lipofectamine RNAiMAX (ThermoFischer scientific) according to manufacturer instructions. 24 hrs post transfection the cells were lysed, and luciferase activity was recorded using Dual-Luciferase® Reporter Assay System kit (Promega). The luciferase signals were measured using Spark® Multimode Microplate Reader (Tecan). Relative Light Units were calculated by normalizing the *Renilla luciferase* signal with *Firefly luciferase* signal.


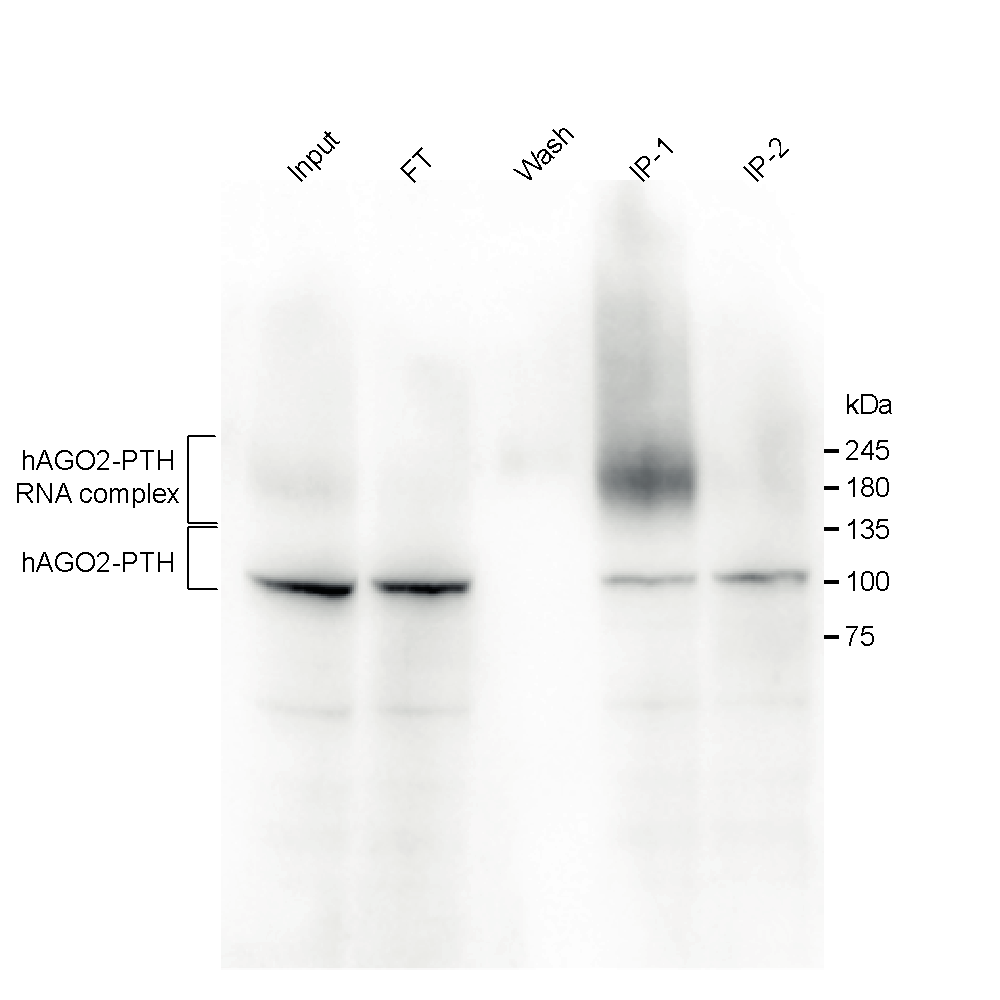


**Supplementary Figure S2:** Western blot analysis of hAGO2-PTH precipitation from HEK293T cells after UV crosslinking. The migration position of AGO2-PTH was detected with AGO2-specific antibodies and is marked on the left. Input is the whole cell lysate, FT is the unbound fraction, W is a high salt wash, IP1 is the elution after the first purification step by a-proteinA antibody, IP2 is elution from the second purification step by Ni-NTA chromatography.

*A full gel image is available as Supplementary Figure S9*


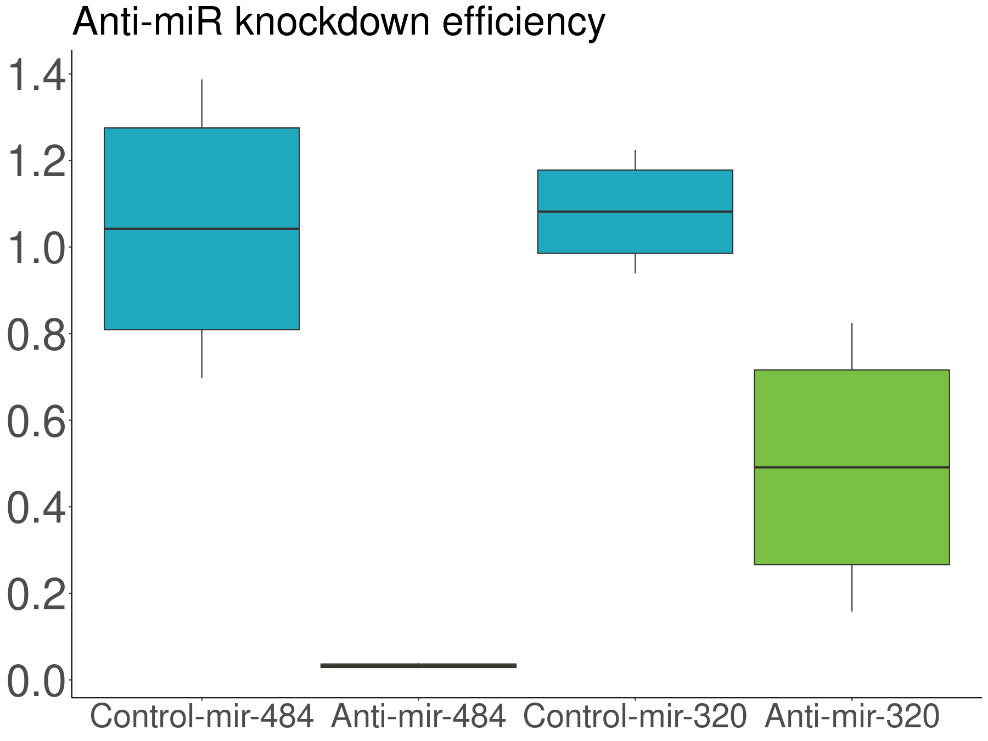


**Supplementary Figure S3**

RT-qPCR quantification of miR-484 ad miR-320a levels in HEK293T cells transfected with anti-miR-484, anti-miR-320a and nontargeting control NC1 (control), respectively. Y axis shows the relative levels of miR-320a and miR484 normalized to endogenous RNU44 RNA.

**Supplementary Table ST3**

List of oligomers, RNA adaptors and synthetic oligonucleotides used in the study. All sequences are written in the 5’ to 3’ orientation.

| **Name** | **Sequence** |
| --- | --- |
|  | **Anti miR probes- synthesized by IDT** |
| antimiR484 | 5'-mA/ZEN/mUmCmGmGmGmAmGmGmGmGmAmCmUmGmAmGmCmCmUmG/3ZEN/-3' |
| antimiR320a | 5'-mA/ZEN/mCmGmCmCmCmUmCmUmCmAmAmCmCmCmAmGmCmUmUmU/3ZEN/-3' |
| NC1 Negative control | 5'-mG/ZEN/mCmGmUmAmUmUmAmUmAmGmCmCmGmAmUmUmAmAmCmG/3ZEN/-3' |
|  | **CLASH linker and primers** |
|  | **3' linker** |
| miRCat-33 adapter 3' | 5'-/5rApp/TGGAATTCTCGGGTGCCAAG/3ddC/-3' |
|  | **5' linkers (barcode in red)** |
| 5P_Linker_UMI | 5'-/5InvddT/ACACrGrArCrGrCrUrCrUrUrCrCrGrArUrCrUrNrNrNrNrNrNrUrArArGrC-3' |
| 5P_Linker_a1_UMI | 5'-/5InvddT/ACACrGrArCrGrCrUrCrUrUrCrCrGrArUrCrUrNrNrNrNrNrNrArUrUrArGrC-3' |
| 5P_Linker_b1_UMI | 5'-/5InvddT/ACACrGrArCrGrCrUrCrUrUrCrCrGrArUrCrUrNrNrNrNrNrNrGrCrGrCrArGrC-3' |
| **PCR primers** |  |
| miRCat-33 primer (R) | CCTTGGCACCCGAGAATT |
| PE_miRCat_PCR (R) | CAAGCAGAAGACGGCATACGAGATCGGTCTCGGCATTCCTGGCCTTGGCACCCGAGAATTCC |
| P5_CLASH (F) | AATGATACGGCGACCACCGAGATCTACACTCTTTCCCTACACGACGCTCTTCCGATCT |
|  | **Luciferase assay primers (in** 5'-3' orientation) |
| NAP1L1 (F) | CAGACTCGAGAAAATGGGTAGTACTGTTTACCTAACTACCTCATGGATGTGTTAAGGCATGCGGCCGCGCAG |
| NAP1L1 (R) | CTGCGCGGCCGCATGCCTTAACACATCCATGAGGTAGTTAGGTAAACAGTACTACCCATTTTCTCGAGTCTG |
| IQCB1 (F) | CAGACTCGAGAGACCTGCAGTAAGCAAAGAGTTATATTCTACCTCTCTCTCAATTTTCTTGCGGCCGCGCAG |
| IQCB1 (R) | CTGCGCGGCCGCAAGAAAATTGAGAGAGAGGTAGAATATAACTCTTTGCTTACTGCAGGTCTCTCGAGTCTG |
| DEGS1 (F) | CAGACTCGAGTAGACACTGCTGAATCCTGTACAGCCTTACTCATAAATAAAGTACTTACTGCGGCCGCGCAG |
| DEGS1 (R) | CTGCGCGGCCGCAGTAAGTACTTTATTTATGAGTAAGGCTGTACAGGATTCAGCAGTGTCTACTCGAGTCTG |
| SLC9A3 (F) | CAGACTCGAGCCATGGCTGGCCGCTCGCGCCCTTTCCCACCGCTGCCGGGAAACCGAGGCGCGGCCGCGCAG |
| SLC9A3 (R) | CTGCGCGGCCGCGCCTCGGTTTCCCGGCAGCGGTGGGAAAGGGCGCGAGCGGCCAGCCATGGCTCGAGTCTG |
| ABCA2 (F) | CAGACTCGAGTGCTGGCCTTTCTCCTGCCCGGCCTCGGAACCAGCTTTTCTCTCTTACGAGCGGCCGCGCAG |
| ABCA2 (R) | CTGCGCGGCCGCTCGTAAGAGAGAAAAGCTGGTTCCGAGGCCGGGCAGGAGAAAGGCCAGCACTCGAGTCTG |
| PRICKLE3 (F) | CAGACTCGAGCCCACCTAAGTCACAAAATGAGGGAAGTGGGGAGTTAGATTTCAGAGTCCGCGGCCGCGCAG |
| PRICKLE3 (R) | CTGCGCGGCCGCGGACTCTGAAATCTAACTCCCCACTTCCCTCATTTTGTGACTTAGGTGGGCTCGAGTCTG |
| PIM3 (F) | CAGACTCGAGAACATGTATAGTGGCTAACTTAAGGGGAGTGGGTGACCCTGACACTTCCAGCGGCCGCGCAG |
| PIM3 (R) | CTGCGCGGCCGCTGGAAGTGTCAGGGTCACCCACTCCCCTTAAGTTAGCCACTATACATGTTCTCGAGTCTG |
| CNBP (F) | CAGACTCGAGCTACATAGAACATACAGTTGAGTGGGAGTAAACAAAAAGATAAACATGCGGCGGCCGCGCAG |
| CNBP(R) | CTGCGCGGCCGCCGCATGTTTATCTTTTTGTTTACTCCCACTCAACTGTATGTTCTATGTAGCTCGAGTCTG |
| SNX11 (F) | CAGACTCGAGGCATTTAGTTCAGAGTGGAGGGGCTTTGGCCTGAAATAAAATGCAAGTATGCGGCCGCGCAG |
| SNX11 (R) | CTGCGCGGCCGCATACTTGCATTTTATTTCAGGCCAAAGCCCCTCCACTCTGAACTAAATGCCTCGAGTCTG |
| HEXB (F) | CAGACTCGAGGAGAACATGTAAAAAATGGAGGGGAAAAAGGCCACAGCAATCTGTACTACGCGGCCGCGCAG |
| HEXB(R) | CTGCGCGGCCGCGTAGTACAGATTGCTGTGGCCTTTTTCCCCTCCATTTTTTACATGTTCTCCTCGAGTCTG |
| PAWR (F) | CAGACTCGAGAACATTTACTGAATGGTGGAGTGAAAAAACTGATGCATACTGGGAAATATGCGGCCGCGCAG |
| PAWR (R) | CTGCGCGGCCGCATATTTCCCAGTATGCATCAGTTTTTTCACTCCACCATTCAGTAAATGTTCTCGAGTCTG |
| CHMP3 (F) | CAGACTCGAGATCTATTTAACCAAGAGGATGCTGGGGAATTGTGCTGCTTGTCCTGTTGGGCGGCCGCGCAG |
| CHMP3 (R) | CTGCGCGGCCGCCCAACAGGACAAGCAGCACAATTCCCCAGCATCCTCTTGGTTAAATAGATCTCGAGTCTG |
| UBTF (F) | CAGACTCGAGCGTTCATGGGGGAGGGGGCAGAAGTGTTTTTTATATATGTGTATATATTTGCGGCCGCGCAG |
| UBTF (R) | CTGCGCGGCCGCAAATATATACACATATATAAAAAACACTTCTGCCCCCTCCCCCATGAACGCTCGAGTCTG |
| DNAJB1 (F) | CAGACTCGAGATCTTGGGGAGGGAAGGGGAGTTGGAACAAATGTCTGTTCCCTGGAGGCCGCGGCCGCGCAG |
| DNAJB1 (R) | CTGCGCGGCCGCGGCCTCCAGGGAACAGACATTTGTTCCAACTCCCCTTCCCTCCCCAAGATCTCGAGTCTG |
| PHC3 (F) | CAGACTCGAGGTGGTGAAAAAGTTGGTGTTTATGAGAGAGTGGTGGGGTGTCTAGTCATTGCGGCCGCGCAG |
| PHC3 (R) | CTGCGCGGCCGCAATGACTAGACACCCCACCACTCTCTCATAAACACCAACTTTTTCACCACCTCGAGTCTG |
| PSMD3 (F) | CAGACTCGAGTAGGGAGTGGGGGACTAGAACTGGGATGTCTTGGCTTGTATGTTTTTTGAGCGGCCGCGCAG |
| PSMD3 (R) | CTGCGCGGCCGCTCAAAAAACATACAAGCCAAGACATCCCAGTTCTAGTCCCCCACTCCCTACTCGAGTCTG |
| ARFGEF1 (F) | CAGACTCGAGGATAAAATGTTTATAATTGGGGAGTGGGGAGAGAAGAAGAAATTATTGTTGCGGCCGCGCAG |
| ARFGEF1 (R) | CTGCGCGGCCGCAACAATAATTTCTTCTTCTCTCCCCACTCCCCAATTATAAACATTTTATCCTCGAGTCTG |
| CDS2 (F) | CAGACTCGAGTCTTGAACTTAGTTTTCTGAATGGGGGTGGGGAGTTGGGGCACGTGTGTGGCGGCCGCGCAG |
| CDS2 (R) | CTGCGCGGCCGCCACACACGTGCCCCAACTCCCCACCCCCATTCAGAAAACTAAGTTCAAGACTCGAGTCTG |
| RAD51 (F) | CAGACTCGAGCTGGACAATCTTATGTTTCCAAGAGAACTAAAGCTGGAGAGACCTGACCCGCGGCCGCGCAG |
| RAD51 (R) | CTGCGCGGCCGCGGGTCAGGTCTCTCCAGCTTTAGTTCTCTTGGAAACATAAGATTGTCCAGCTCGAGTCTG |
| HIST1H2BJ (F) | CAGACTCGAGAAACAGTGAGTTGGTTGCAAACTCTCAACCCTAACGGCTCTTTTAAGAGCGCGGCCGCGCAG |
| HIST1H2BJ (R) | CTGCGCGGCCGCGCTCTTAAAAGAGCCGTTAGGGTTGAGAGTTTGCAACCAACTCACTGTTTCTCGAGTCTG |
| CCND2 (F) | CAGACTCGAGCAAGAAGGAATCCTGGATTTTGCCCTCTCCTTAGCTCTTAGTCTCTTTGGGCGGCCGCGCAG |
| CCND2 (R) | CTGCGCGGCCGCCCAAAGAGACTAAGAGCTAAGGAGAGGGCAAAATCCAGGATTCCTTCTTGCTCGAGTCTG |
| YOD1 (F) | CAGACTCGAGGGAGAAGTGTGACCTATGCATGAATGAGGGTTGAAGCCTACTACCTCACAGCGGCCGCGCAG |
| YOD1 (R) | CTGCGCGGCCGCTGTGAGGTAGTAGGCTTCAACCCTCATTCATGCATAGGTCACACTTCTCCCTCGAGTCTG |
| UCK2 (F) | CAGACTCGAGTCTGGGTGGAGGGGAGCCCAAGTGAGCCTGAAGGAGTTCTGATTATAGAGGCGGCCGCGCAG |
| UCK2 (R) | CTGCGCGGCCGCCTCTATAATCAGAACTCCTTCAGGCTCACTTGGGCTCCCCTCCACCCAGACTCGAGTCTG |
| TOP1 (F) | CAGACTCGAGATAAACCTGGAGATATTATAAGGGAGAGCTGAGCCAGTTGTCCTATGGACGCGGCCGCGCAG |
| TOP1 (R) | CTGCGCGGCCGCGTCCATAGGACAACTGGCTCAGCTCTCCCTTATAATATCTCCAGGTTTATCTCGAGTCTG |
| SCN1B (F) | CAGACTCGAGTGCCCCGGCCCAACCTCGCCCTCTCTCACCAGCCTTGAACTGTGGCCACCGCGGCCGCGCAG |
| SCN1B (R) | CTGCGCGGCCGCGGTGGCCACAGTTCAAGGCTGGTGAGAGAGGGCGAGGTTGGGCCGGGGCACTCGAGTCTG |
|  | **Taqman assay oligos** |
| Hsa-miR-484  assay ID: 001821 | 5'-UCAGGCUCAGUCCCCUCCCGAU-3' |
| Hsa-miR-320a  assay ID: 002277 | 5'-AAAAGCUGGGUUGAGAGGGCGA-3' |
| Hsa-miR-92a-3p  assay ID: 000431 | 5'-UAUUGCACUUGUCCCGGCCUGU-3' |
| RNU44  Assay ID: NR_002750 | - |

**Bioinformatics**

**1. Method overview**

The CLASH (Cross-Linking, Ligation, and Sequencing of Hybrids) protocol generates reads composed of two distinct interacting RNA molecules that have been partially digested and joined by intermolecular ligation at one end. At both ends of molecular construct the adaptors necessary for high throughput sequencing are added. To be able to conclusively determine PCR duplicates introduced during the library preparation for sequencing, the 5’ end adapter is enriched with Unique Molecular Identifiers (UMI).

Our bioinformatics analysis solution begins with UMI extraction, detection and trimming of sequencing adapters as well as filtering of low quality data.

As a next step after preprocessing we align all reads passing the previous step onto the whole human genome using very strict alignment settings to identify single genomic reads. Mapping to the whole genome gives us an outstanding opportunity to obtain all uncommon and purely described interactions within introns or even in intergenic regions. All reads uniquely aligned to the human genome are separated and considered as non-chimeric apart from those originating from our masked regions. Masked region consists of all non-coding RNAs belonging to one of the following biotypes: miRNA, rRNA, tRNA, snoRNA, YRNA and vaultRNA.

All of the unmapped reads together with reads from masked regions subject at another round of alignment to every single non-coding RNA list mentioned before, serving to determine both the single non-coding reads and chimeric candidates. Reads fully covering any non-coding RNA without any significant sequence overhang are separated and considered as single non-coding RNA non-chimeric reads. Reads partially covering any non-coding RNA and at the same time showing significant sequence overhang are moved further and considered as potential chimeric candidates.

Unmapped overhang parts of reads obtained after mapping to individual non-coding RNA databases are further mapped to the whole genome. Perfectly mapped overhang sequence reads determine the genomic part of potential chimeric reads.

After the chimeras are called, all duplicated records are removed using UMI’s and postprocess filters i.e. no mismatch in alignments, support of chimeric alignment by single reads alignment, alignment lengths of individual chimeric read parts, etc. may be applied to further refine called chimeras. To reduce the redundancy of called chimeras we collapsed all chimeras from specific non-coding RNA targeting the same genomic region with some minor discrepancy into one chimera.

Next to the filters, interactions and base pairing within every single called chimera is predicted by folding the chimeric sequence. At the same time RepeatMasker annotation and number of unique and duplicated reads supporting individual chimera is determined.

Whole executable pipeline together with all codes and databases can be found at <https://github.com/ML-Bioinfo-CEITEC/HybriDetector>.

**2. CLASH analysis pipeline**

**2.1.  Preprocessing reads**

High-throughput CLASH data were generated using the NEBNext Ultra II Directional RNA Library Prep Kit for Illumina, featuring polyA selection, and sequenced on an Illumina NextSeq sequencer (run length 1x75 nt). Bcl files were converted to Fastq format using bcl2fastq v. 2.20.0.422 Illumina software for base calling. Unique molecular identifiers (UMIs) with a length of 6 nucleotides were extracted and subsequently employed for deduplication of aligned reads using UMI-tools v. 1.1.1 [3] .The quality of raw Fastq reads was assessed using FastQC v. 1.7 [4].

Preprocessing steps are contingent on the experimental design, which influences the sequenced read's scope, whether it includes random nucleotide sequences corresponding to UMIs, or the adapter sequences specific to individual sequencing library preparation kits. To address these inconsistencies, users can choose to extract the UMI sequence when present or not.

Given the nature of CLASH construct preparation, it is common for recovered fragments to be shorter than the actual sequenced read length. This leads to the presence of adapter sequences within the read sequence. It is essential to accurately remove all adapter artifacts from the read sequence to prevent bias and to accurately call chimeric reads.

To detect sequenced barcodes, we utilized the minion tool from the Kraken toolset [5] to identify all overrepresented subsequences in the Fastq file. In a second round, we employed the swan tool from the same Kraken toolkit to annotate, by comparison to our in-house adapter sequence database, whether the overrepresented sequences might originate from library preparation chemistry or are merely PCR artifacts.

After identifying the adapters, we used the Trimmomatic tool [6] with settings ILLUMINACLIP: ’TGGAATTCTCGGGTGCCAAG’, ‘AGATCGGAAGAGCGGTTCAG’:2:30:5:3:true CROP:75 LEADING:3 TRAILING:3 SLIDINGWINDOW:4:5 MINLEN:16 to remove all possible adapter sequences from each sequencing read, while also removing low-quality bases or entire reads. All reads with resulting sequences shorter than 16 nucleotides after trimming were discarded and removed from the dataset, and the same applied to all trimmed nucleotides from individual reads.

**2.2.  Mapping reads to obtain single genomic reads**

**2.2.1 Index generating**

Typical mapping software requires a precomputed index of the reference genome for efficient and accurate assignment of sequenced reads to their regions of origin. For each reference database, either built-in or user-provided, the index is computed using the STAR [7] software with the setting --runMode genomeGenerate.

**2.2.2 Mapping to reference genome**

CLASH-recovered RNA fragments may contain a large number of molecules where intermolecular ligation has failed. As a result, part of the retrieved fragments serves as a standard RNA-Seq sequence library. To achieve precise genomic mapping of non-ligated RNA fragments, we adopted STAR, the gold standard of RNA-Seq analysis aligners.

Single genomic reads must consist of a continuous sequence that maps to a specific genomic region, with any unaligned portion being significantly short. To meet these requirements, we used STAR with default settings and modified the following parameters:

- --outFilterMismatchNoverReadLmax 0.1
- --outFilterMismatchNoverLmax 0.1
- --outFilterScoreMinOverLread 0.85
- --outFilterMatchNminOverLread 0.85

In other words, at least 85% of the trimmed sequenced read must be fully aligned, allowing for only a number of mismatches equal to 10% of the read length. We further separated all reads aligned to the genomic regions of all our non-coding RNA database targets while filtering out any alignments spanning junctions. For more accurate quantification of genomic coverage derived from single reads, we used only uniquely mapped reads.

All reads identified as single genomic were filtered from the dataset and quantified separately.

**2.3.  Mapping reads to obtain single non-coding RNA reads**

**2.3.1 Non-coding RNA database**

Depending on the number of potential chimeric target biotypes detected, it is important to focus on collecting appropriate databases to prepare a sufficiently comprehensive search space. In our pipeline, independent databases are available for the following non-coding RNA biotypes: rRNA, tRNA, miRNA, YRNA, snoRNA, and vaultRNA.

Despite improvements in the annotation and knowledge of specific non-coding RNA biotypes over the past years, finding a comprehensive source containing all known annotations for even a single non-coding RNA biotype remains challenging. We chose to gather annotations from multiple sources to create as comprehensive databases as possible.

1. For **rRNA**, we identified four sources of annotation:
   1. From **NCBI**, we downloaded the Homo Sapiens RefSeq reference genome annotation and filtered the records corresponding to rRNA annotation.
   2. From **Ensembl**, we downloaded the Homo Sapiens reference genome annotation and filtered the records corresponding to rRNA annotation.
   3. From **UCSC**, we downloaded the RepeatMasker annotation and filtered the records corresponding to rRNA.
   4. From the **SILVA** database, we downloaded fasta sequences of ribosomal Small and Large subunits. Fasta files were cleaned of duplicated records. Downloaded fasta files were mapped against the human hg38 reference genome to obtain genomic locations of rRNA sequences.
2. For **tRNA** we found 5 sources of annotation:
   1. From **NCBI**, we downloaded the Homo Sapiens RefSeq reference genome annotation and filtered the records corresponding to tRNA annotation.
   2. From **Ensembl**, we downloaded the Homo Sapiens reference genome annotation and filtered the records corresponding to tRNA annotation.
   3. From **UCSC**, we downloaded the RepeatMasker annotation and filtered the records corresponding to tRNA.
   4. From **UCSC**, we downloaded the Genes and Gene Predictions track filtered to only tRNA from Homo Sapiens.
   5. From the **GtRNAdb** database, we downloaded all tRNA fasta sequences with genomic locations specific for Homo Sapiens.
3. For **YRNA** we found 5 sources of annotation:
   1. From **Ensembl** we downloaded the Homo Sapiens reference genome annotation and filtered the records corresponding to YRNA annotation.
   2. From **UCSC** we downloaded the RepeatMasker annotation and filtered the records corresponding to just YRNA.
   3. From **UCSC** we downloaded Genes and gene predictions track filtered to only YRNA from Homo Sapie
4. For **miRNA**, we used the miRBase mature miRNA collection as a database.
5. As a **vaultRNA** database we adopted annotations from UCSC.
6. We parsed the Dashr database to obtain information related to **snoRNA.**

**2.3.2 Mapping to non-coding RNA database**

After all noncoding RNA databases were cleaned and standardized to the same format, we used them as reference databases and mapped all of the sequenced reads, excluding those separated as described in section 2.2.2. We aligned sequenced reads using the STAR aligner with default settings and modified the following parameters:

- --outFilterMismatchNoverReadLmax 0.1
- --outFilterMismatchNoverLmax 0.1
- --outFilterScoreMinOverLread 0.25
- --outFilterMatchNminOverLread 0.25

Allowing for alignment of at least 25% of the read length, combined with subsequent filtering steps, provides a powerful opportunity to identify either single noncoding RNA reads or chimeric reads in a single alignment step.

Sequenced reads were aligned separately to individual databases to prevent misalignments. If all noncoding RNA databases were merged into a single archive, the resulting database would contain duplicated records either in terms of genomic locations or genomic sequences. For example, a sequence from miRNA might resemble a small part of rRNA or tRNA, even if they originate from different genomic locations. This could lead to misalignments or misannotations, where the read would be aligned to the first suitable sequence or reported as mapped to multiple reference sequences.

**2.3.3 Harvesting of single non-coding RNA reads**

It is essential to accurately identify and annotate the non-coding RNA fragment part of a chimeric read to avoid incorrect interpretations. Our goal of obtaining the most precise annotated chimeric reads led to multiple alignments of a single sequenced read onto more than one non-coding RNA target. The procedure above results in large alignment files for all individual noncoding RNA databases, containing both single noncoding RNA reads and potential chimeric reads.

To reduce the dataset's complexity, we filtered out all unmapped reads, reads mapped to the reverse strand, and all supplementary alignments. To accurately determine mapping to specific genomic locations, we filtered out all reads shorter than 18 nt since this is the shortest record across our noncoding RNA databases. Additionally, the chance of uniquely specifying the read's genomic location of origin significantly decreases with sequences shorter than 18 nt. At the same time, we discarded all reads with an aligned part shorter than 15 nt for the same reason.

We concatenated the resulting alignment files from all alignments to individual non-coding RNA databases and sorted the file by sequenced read name. This provides us a block of all possible alignments from individual references for each sequenced read.

Single noncoding RNA reads were designated as reads with only one alignment within the block or with more alignments of the same sequence to multiple references. These reads have an aligned matched part longer than 15 nt and do not contain more than 6 nt of unmapped sequence from both ends of the reads (soft-clipped part).

**2.4 Seeking chimeric reads**

**2.4.1 Selecting potential chimeric reads and definition of noncoding chimeric part**

Chimeric reads are defined as reads composed of two different RNA fragments originating from distinct genomic regions. For each block of alignments to our selected noncoding RNA databases for every single read described above containing more than one alignment, we applied the following logic:

1. Identify the matching part of each read (M and I characters).
2. Calculate the union and intersection length of matches.
3. If the intersection length (overlap of pairwise alignment) is > 80% of the union length (sum of pairwise alignment), the reads are considered similar enough.
4. If that is not the case, the whole block is discarded (these are reads that have different parts matching).
5. For reads that are similar enough, the read with the longest S part is selected as representative of the block.
6. The reference name of the representative read has the references of the other members of the block appended with "|" (e.g., miR-432|snorna-1).

In cases where one block contains two different alignments to distinct records in databases, each aligned part is at least 15 nt long, the soft-clipped part is longer than 6 nt, and the intersection length (overlap of pairwise alignment) is at most 4 nt, we consider these reads as "reference:reference" or "small:small chimeric" reads. Reads with 15 or more S on one side and 6 or less S on the other are marked as 'potentially chimeric'. Next, the longer soft-clipped unaligned part of the read sequence is extracted from the potentially chimeric read and further examined. All other alignment blocks are discarded, as these are ambiguous and it is difficult to determine the origin of such reads.

**2.4.2 Selecting potential chimeric reads**

Sequences of soft-clipped parts longer than 15 nucleotides were converted to a fastq file and underwent a second round of alignment against the entire human genome without any genome masking applied. In this alignment step, we sought AGO2 genomic target locations specified within the chimeric read sequence. We again utilized STAR with default settings and changed the following parameters:

--outFilterMismatchNoverReadLmax 0.1

--outFilterMismatchNoverLmax 0.1

--outFilterScoreMinOverLread 0.75

--outFilterMatchNminOverLread 0.75

By using stricter settings for aligned length, we achieve higher precision in determining genomic targets interacting with their guides. Only uniquely mapped soft-clipped parts were further processed, and reads spanning any junctions were discarded.

**2.4.3 Annotation GTF file modification**

To obtain a more specific and non-redundant annotation of target parts of chimeric reads, we modified the Ensembl v94 GTF annotation. Redundancy in the annotation is a consequence of the multiple differently annotated transcripts within a GTF file, leading to overlapping annotations, particularly within the starting or ending exons and untranslated regions on both sides. We addressed this by selecting the longest transcript for each annotated gene, where both untranslated regions were defined as a union of all annotated untranslated regions across available transcripts for a particular gene, separately for 3'UTR and 5'UTR.

**2.4.4 Connection, deduplication and annotation of potential chimeric reads**

After both guide and target parts of chimeric reads are individually defined, they are reconnected based on the read name, and the order of whether the guide or target were first in the chimeric read is noted. Chimeras with guide parts aligned to the reverse strand as well as all non-primary alignments, were removed.

All detected potential chimeric reads were deduplicated based on the Unique Molecular Identifier (UMI) sequence and the loci of the target part alignment, as well as the sequence of the read, while the amount of duplication remains tracked as records about the number of duplicated and unique reads.

A modified GTF file (see section 2.4.3) from Ensembl v94 was used for the gene name and gene feature annotation of the target part of the chimeric read. From the SAM flag, the strand of the target part alignment was derived, and a RepeatMasker database downloaded from UCSC on 21.5.2021 was utilized to identify target part alignments within repetitive regions and the family of the repeat.

Single genomic non-chimeric reads were deduplicated using UMIs by utilizing UMI-tools [3] and followed by deepTools bamCoverage [8] to obtain bedgraph files containing the read coverage of single genomic reads across the genome. Coverage tracks were employed to report whether the obtained target part of the chimeric read was supported by the alignment of single genomic non-chimeric reads and also the level of support.

In cases where the guide part originated from miRNA, the miRNA family is annotated together with the information if the obtained guide sequence from the chimeric read is perfectly aligned to the reference guide sequence; this is marked as alignment without any mismatches. Next, the complexity of the guide sequence is evaluated, and the percentage representation of each nucleotide is noted.

In the end, we gathered the chimeric sequences and utilized RNAcofold in default settings from the ViennaRNA package [9] to predict the ability of the sequence to cofold the secondary structure. In the case of miRNA, the database sequence was used as input; for the rest of the guide types, the original obtained sequence was used. Both the depicted secondary structure in dot-bracket notation and the predicted Minimum Folding Energy (MFE) are assigned to each chimera.

**2.4.5 Filtering and collapsing of potential chimeric reads**

To reach the higher level of certainty of discovered chimeric reads the potential chimeric reads are filtered to meet the following cutoffs:

1. Alignment of the target genomic part has to be at least 20 nt long
2. Target part of the genomic read has to be supported by at least one single genomic read
3. Guide part of the read has to be complex enough, each nucleotide type has to overcome at least 50 percent of abundance across the guide sequence
4. Guide part alignment cannot be longer than 30 nt as biologically it would be difficult for AGO2 to load longer guide sequences

 During the whole process of CLASH sequencing library preparation as well as during the sequencing process itself there is a high probability of introduction of errors or single nucleotide polymorphism originating from the technical shortcomes of the technology. This gives rise to a number of very similar sequences differing in only from 1 to a few nucleotides but originally coming from the same cell of origin.

First the target parts of reads are collapsed together by sequence where the guide sequence remain uncollapsed, the most abundant read is chosen as representative one, the target part is defined as length union of all target parts starting at the same genomic position and the abundancy and support by single genomic read is summed across reads collapsed together. This new record is called “chimeric interaction” and contains the representative most abundant obtained chimera as well as collapsed derived chimera.

Second the target parts of chimeric interactions are collapsed together whenever there are two interactions with the same guide part and target parts starting within the surrounding of 20 nt next the the other defined as union length of target parts.

Next the single nucleotide polymorphisms as well as other minor sequence differences are resolved using the hierarchical clustering of the chimeric interaction sequences to obtain a unique list of chimeric interactions. The clustering was performed as following:

1. First the generalized Levenshtein (edit) distance, giving the minimal possibly weighted number of insertions, deletions and substitutions needed to transform one string into another is computed
2. Second the editing distance is normalized to the mean length of two compared sequences to avoid bias introduced by the length of the compared sequences
3. Hierarchical clustering of normalized editing distance is performed
4. The hierarchical tree is cut at the height of 0.3 where all of the branches below this value are treated as a members of the same cluster

Chimeric interactions are then collapsed based on the cluster they belong to and the most abundant interaction is picked as the representative one, while the numbers of reads as well as number of supporting single genomic reads are again summed up. As a last step the target part of the collapsed chimeric interactions are derived from the reference genome in a length of full union of all reads included within a particular interaction and afterwards the center of the target part is identified and the target part of chimeric interaction is defined as +- 25 nt around the defined center to make sure, that whole interacting target site is included within chimeric interaction. Whole chimeric interaction is reannotated in the same way as described in a section above.

In case there are more replicate samples, they are bound together, sorted, collapsed, clustered and reannotated using the same algorithm logic. Based on the fact if the guide part of chimeric interaction aligned perfectly to the reference record there are introduced two categories of the chimeric interactions - low confident and high confident.

**2.4.6 Resolving of reference:reference chimeric reads**

Chimeric reads reporting more than one 15 nt alignment on the distinct part of the read to a different reference record and having at most 4 nt overlap are marked as reference:reference chimeras.

All detected refref chimeras were processed in the similar way as chimeric. First were deduplicated based on the Unique molecular identifier and read sequence. Second both parts of the read were individually collapsed with the chimeras in 20 nt surroundings with respect to the record on the other side of the read. After the hierarchical clustering based on normalized editing distances was performed on both parts of refref chimera separately. All of the refref chimeras with low sequence complexity where any of both chimeric parts contained more than 50% of nucleotide of one type within a sequence were discarded. Cofold prediction of the secondary structure together with Minimum folding energy were added to every refref interaction and based on the perfect alignment of both parts the high confidence and low confidence chimeras are defined.

**3. Drosha CLIP-seq analysis**

Drosha CLIP-Seq data were donwlaoded from GEO database under accession number GSE93651. High-throughput pair-end CLIP-Seq data of DROSHA were quality checked by FastQC v0.11.9 [4]. The adapters and quality trimming of raw fastq reads was performed using Trimmomatic v0.36 [6] with settings CROP:250 LEADING:3 TRAILING:3 SLIDINGWINDOW:4:5 MINLEN:35 and adaptor sequence ILLUMINACLIP:'CAGAGTTCTACAGTCCGACGATC', 'TGGAATTCTCGGGTGCCAAGGAACTCCAGTCAC' and

'GATCGTCGGACTGTAGAACTCTGAACGTGTAGATCTCGGTGGTCGCCGTATCATTAAAAAAAAA'. Trimmed CLIP-Seq reads were mapped against the human genome (hg38) and Ensembl GRCh38 v.94 annotation using STAR v2.7.3a [7] as splice-aware short read aligner and default parameters except --outFilterScoreMinOverLread 0.5, --outFilterMatchNminOverLread 0.5, --outFilterMismatchNoverLmax 0.1 and --twopassMode Basic. Quality control after alignment concerning the number and percentage of uniquely- and multi-mapped reads, rRNA contamination, mapped regions, read coverage distribution, strand specificity, gene biotypes and PCR duplication was performed using several tools namely RSeQC v2.6.2 [10], Picard toolkit v2.18.27 [11] and Qualimap v.2.2.2 [12] and BioBloom tools v 2.3.4-6-g433f [13]. Aligned BAM files were converted into bedgraphs by using deepTools bamCoverage v3.5.1 [8] and visualized in IGV genome browser.


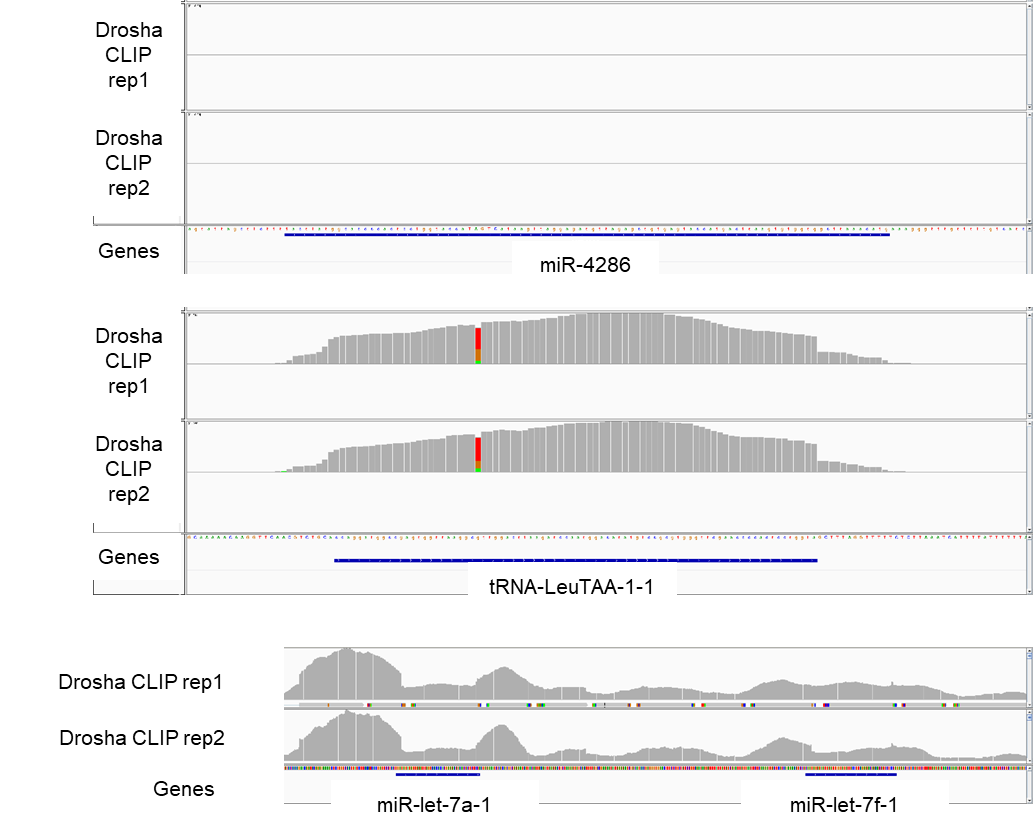


**Supplementary Fig. S4:** Drosha CLIP coverage of HEK293T cells for (top to bottom): hsa-miR-4286 locus, tRNA-Leu-TAA-1-1 locus, hsa-let-7a locus showing coverage for hsa-let-7a (positive control) and tRNA-Leu-TAA-1-1 but not hsa-miR-4286.

**4. Quant-Seq validation of the impact of selected miRNA**

High-throughput RNA-Seq data of miRNA anti-miR knockdown assay were prepared using Lexogen QuantSeq 3' mRNA-Seq Library Prep Kit FWD for Illumina with polyA selection and sequenced on Illumina NextSeq 500 sequencer (run length 1x75 nt). Bcl files were converted to Fastq format using bcl2fastq v. 2.20.0.422 Illumina software for basecalling. 6-nt long UMIs were extracted and subsequently used for deduplication of aligned reads by UMI-tools v. 1.1.1. As a next step 6-nt long barcode sequence related to Lexogen QuantSeq Library Prep Kit were trimmed using seqtk 1.3-r106 [14]. Quality check of raw single-end fastq reads was carried out by FastQC v0.11.9 [4]. The adapters and quality trimming of raw fastq reads was performed using Trimmomatic v0.36 [6] with settings CROP:250 LEADING:3 TRAILING:3 SLIDINGWINDOW:4:5 MINLEN:35 and adaptor sequence ILLUMINACLIP:AGATCGGAAGAGCACACGTC. Trimmed RNA-Seq reads were mapped against the human genome (hg38) and Ensembl GRCh38 v.94 annotation using STAR v2.7.3a [7] as splice-aware short read aligner and default parameters except --outFilterMismatchNoverLmax 0.1 and --twopassMode Basic. Quality control after alignment concerning the number and percentage of uniquely- and multi-mapped reads, rRNA contamination, mapped regions, read coverage distribution, strand specificity, gene biotypes and PCR duplication was performed using several tools namely RSeQC v2.6.2 [10], Picard toolkit v2.18.27 [11] and Qualimap v.2.2.2 [12] and BioBloom tools v 2.3.4-6-g433f [13].

The differential gene expression analysis was calculated based on the gene counts produced using featureCounts tool v1.6.3 [15] with settings -s 2 -T 10 -F GTF -Q 0 -d 1 -D 25000 and using Bioconductor package DESeq2 v1.20.0 [16]. Data generated by DESeq2 with independent filtering were selected for the differential gene expression analysis to avoid potential false positive results. Only genes with overall baseMean expression >= 2 were considered to draw log2(fold-change) density plots. Whole set of gene expressions was divided into two lists based on the evidence of individual genes within a detected chimeric interaction of specific microRNA. To assess the statistical significance of the log2(fold-change) values difference between two lists (occurring within the observed miRNA-mRNA interactions vs non-interacting), Wilcoxon one-sided ranksum test was performed. Supplementary Figure S5 produced using ggplot v3.3.3 package [17].


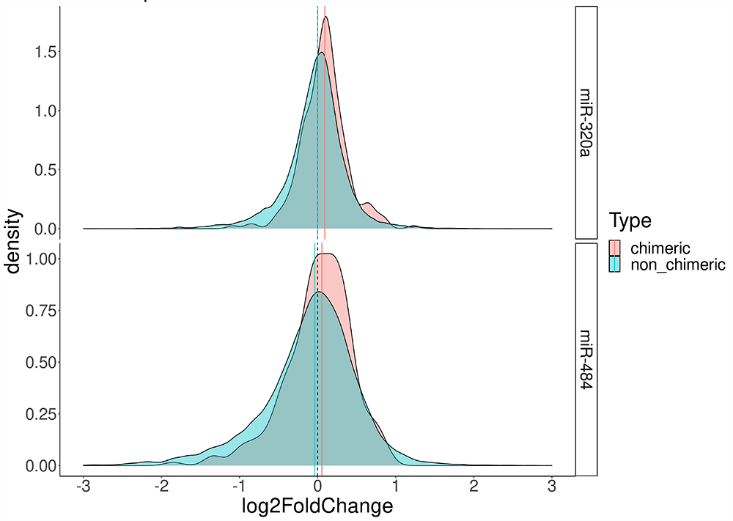


**Supplementary Fig. S5**

Density plot of log2(fold-change) values of two gene lists (occurring within the observed miRNA-mRNA interactions vs non-interacting), Wilcoxon one-sided ranksum test. miR-320a (Wilcoxon Rank Sum Test p=2e-06), miR-484 (Wilcoxon Rank Sum Test p=0.0022).

**5. Luciferase Assay validation of the impact of specific miRNA targets**

Gathered intensities of Luciferase reporter assays were adopted in a form of Renilla/Firefly ratios. Each measurement was performed in quadruplicates. If the ratio *Mean / Standard deviation of the measured quadruplicates* was *<= 5*, the measurement series was further processed to remove outliers. Renilla/Firefly ratio measurement was defined as an outlier when its value was  >=  *Mean + Standard deviation* or *<=* *Mean - Standard deviation*. Such measured Renilla/Firefly ratio was not considered in resulting graphs. Both treatment measurement types (*target + guide* and *target + negative vector*) were normalized to the average measurement of *target only* experiments. In the end all of the *target + guide* measures were divided by the average of *target + negative vector* measures to anchor measurements without any effect at the level of 1. Normalised average *target + guide* measurements together with their standard error were used to produce barplots. Plots were produced using ggplot v3.3.3 package [17].

**6. Convolutional Neural Network**

We trained a Convolutional Neural Network based on the architecture described in [18]. Four different models were trained based on four different datasets introduced in this paper: tRNA, YRNA, whole mature miRNA and truly observed miRNA. The positive samples were taken from the corresponding experimental datasets after bioinformatical cleaning. They were further processed by cutting small RNA sequences from the beginning to the length of 20 bp and splitting them into train and test sets based on the target chromosome number. The negative sets were formed by matching randomly selected small RNA from the positive set and randomly selected target from the positive set, taking care that the selected pair is not present in the positive set. The final training set was composed of a positive: negative ratio of 1:10, and the test sets had 1:1, 1:10 and 1:100 ratios.

To encode the input small RNA - target pairs, we build a 20 (small RNA size) x 50 (target size) matrix in which any Watson-Crick binding nucleotide pair is represented by 1, and any nonbinding pair or empty space in shorter sequences by 0.

The CNN architecture consists of 6 layered blocks composed of a convolutional layer, leaky ReLU, batch normalization, pooling and dropout layer. The output of the last dropout layer is flattened and connected to 2 layered blocks of dense, leaky ReLU, batch normalization and dropout layer. The last layer is formed of a single neuron with a sigmoid activation function, which outputs the probability of input small RNA: target site binding. The network was compiled with Adam optimizer, binary cross-entropy loss function was used. The models were trained over 10 epochs, with a batch size of 32. Convolutional layers had kernels sized 5 × 5, the dropout rate in the dropout layers was 0.3, and the learning rate was 0.00152.

All codes and datasets used for training and testing the method together with python script and user-friendly web server for custom predictions of small RNA mRNA target pair binding probability can be found at <https://github.com/ML-Bioinfo-CEITEC/HybriDetector/tree/main/ML>.


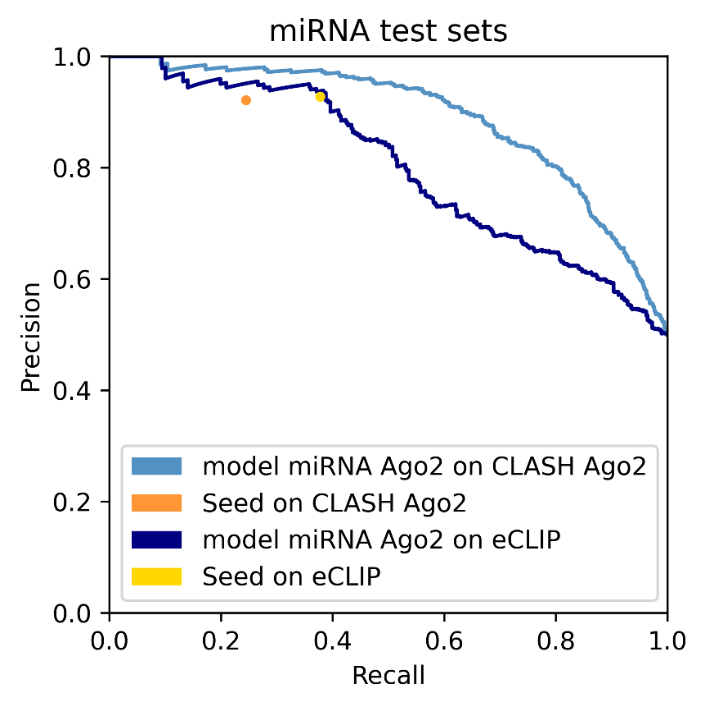


**Supplementary Figure S6**: Precision Recall curve of CNN model trained on AGO2-CLASH and evaluated on left out miRNAs from AGO2-CLASH, and on AGO2-eCLIP data.

| Area under Precision-Recall curve | 1:1 test set | 1:10 test set | 1:100 test set |
| --- | --- | --- | --- |
| Our Model  CNN (AGO2) | **0.8891** | **0.6058** | **0.2178** |
| miRBind (trained on AGO1) | 0.8535 | 0.5167 | 0.1564 |
| Cofold | 0.7709 | 0.2896 | 0.0394 |
| RNA22 | 0.6884 | 0.2151 | 0.0311 |

Supplementary Table ST4 – comparison of methods on miRNA chimeric read test set at three levels of positive:negative class imbalance


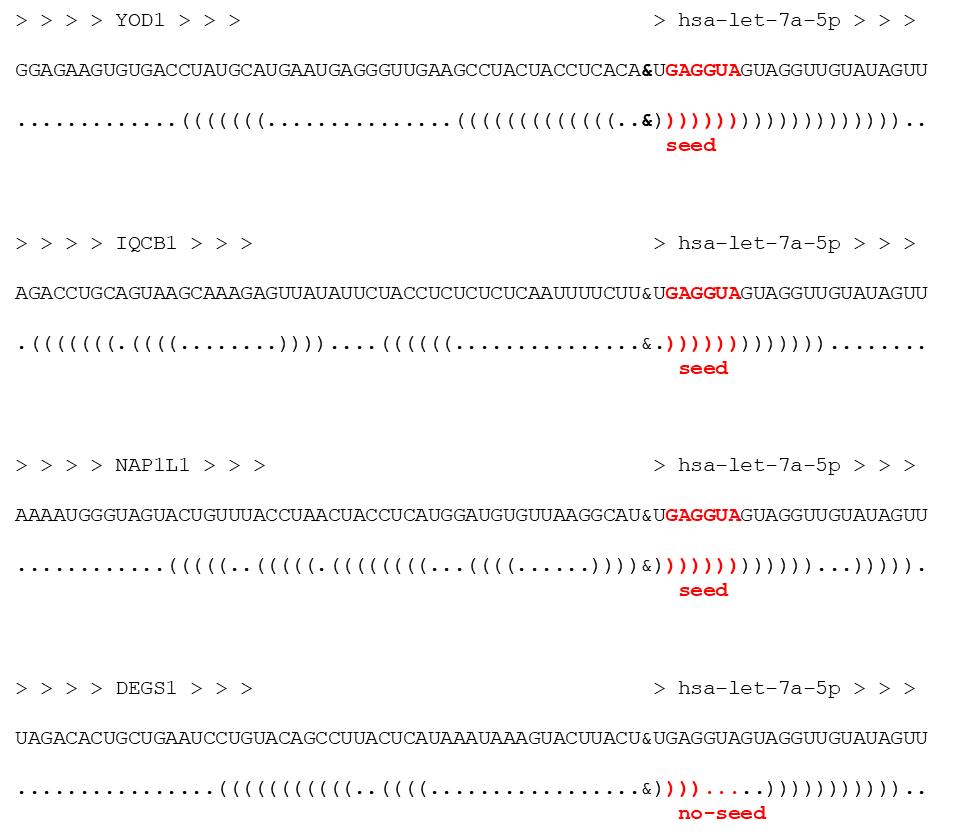


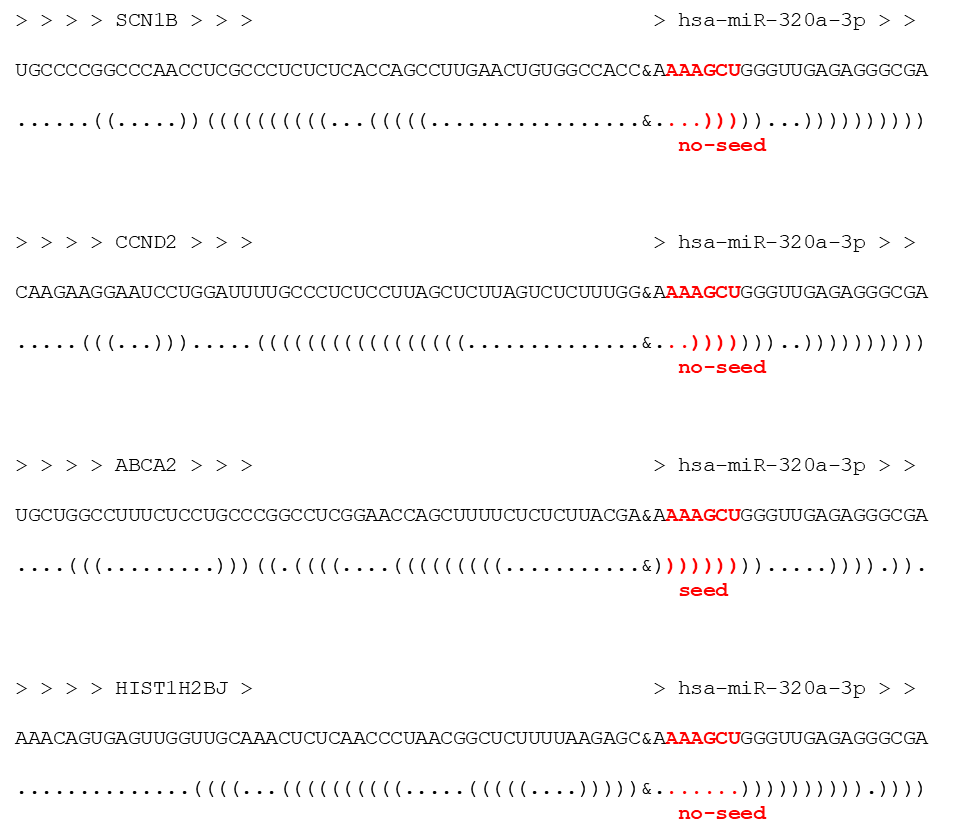


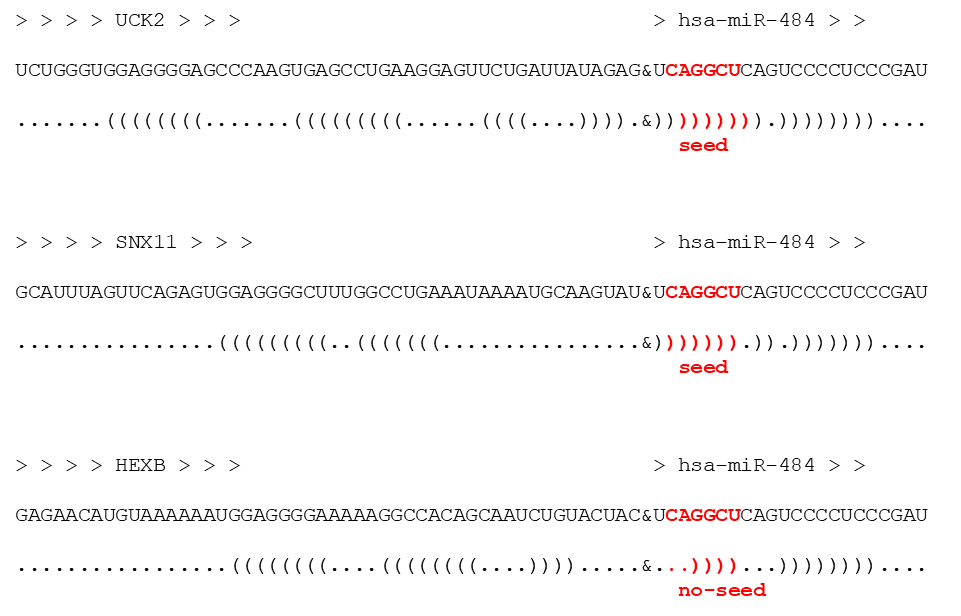


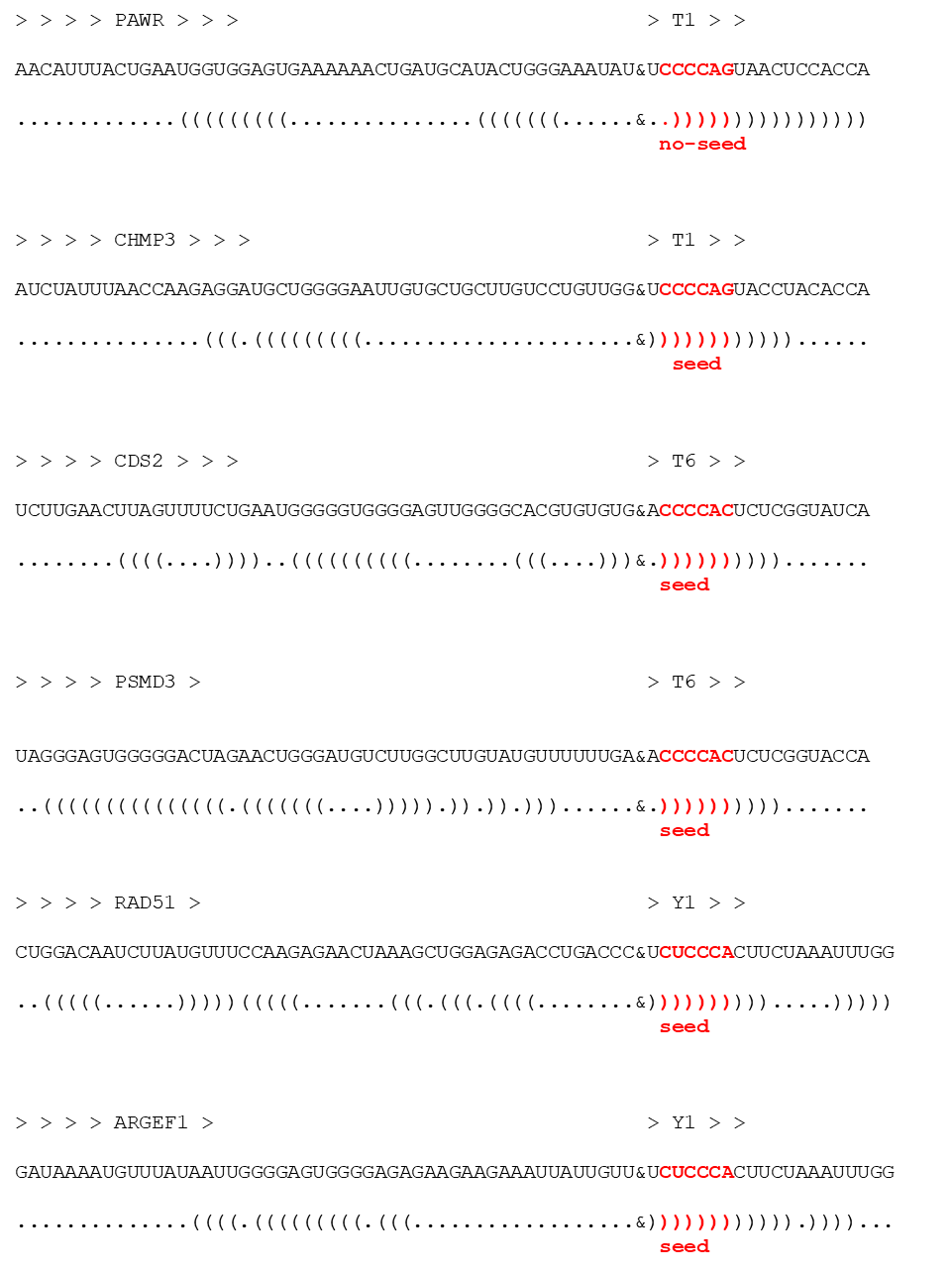


**Supplementary Figure S7:** Schematic RNA cofold representation of all guide:target pairs tested with Luciferase assay.


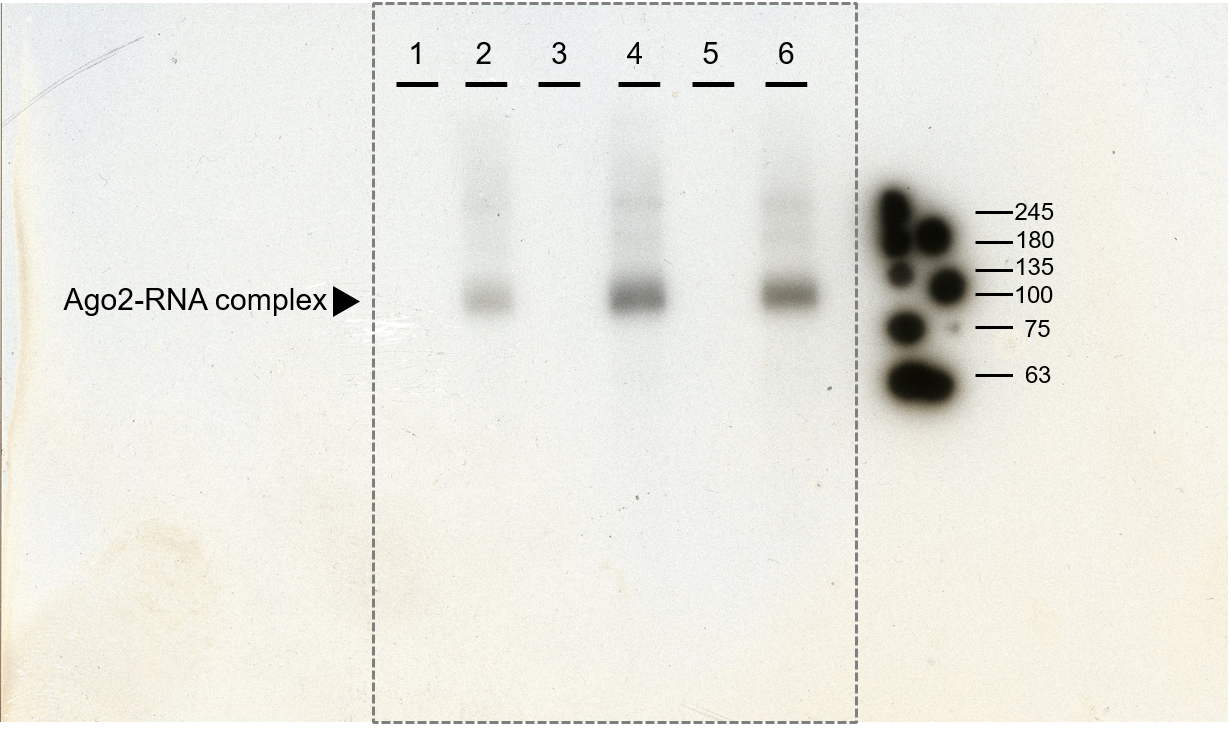


**Supplementary Figure S8:** Raw full gel image of *Supplementary Figure S1*

Autoradiography of 32P-labeled RNA–protein complexes resolved by SDS-PAGE (4–20% gradient gel). Protein-RNA complexes were transferred to the nitrocellulose membrane, dried between Whatmann filter paper no 3 and exposed to Xray film (Cytiva Amersham hyperfilm (Cytiva 28-9068-43) for 1 hour in an X-ray film cassette at Room temperature and developped using a standard Xray film developer. The X-ray film was scanned using an Epson V600 scanner using the manufacturer's software and default settings.


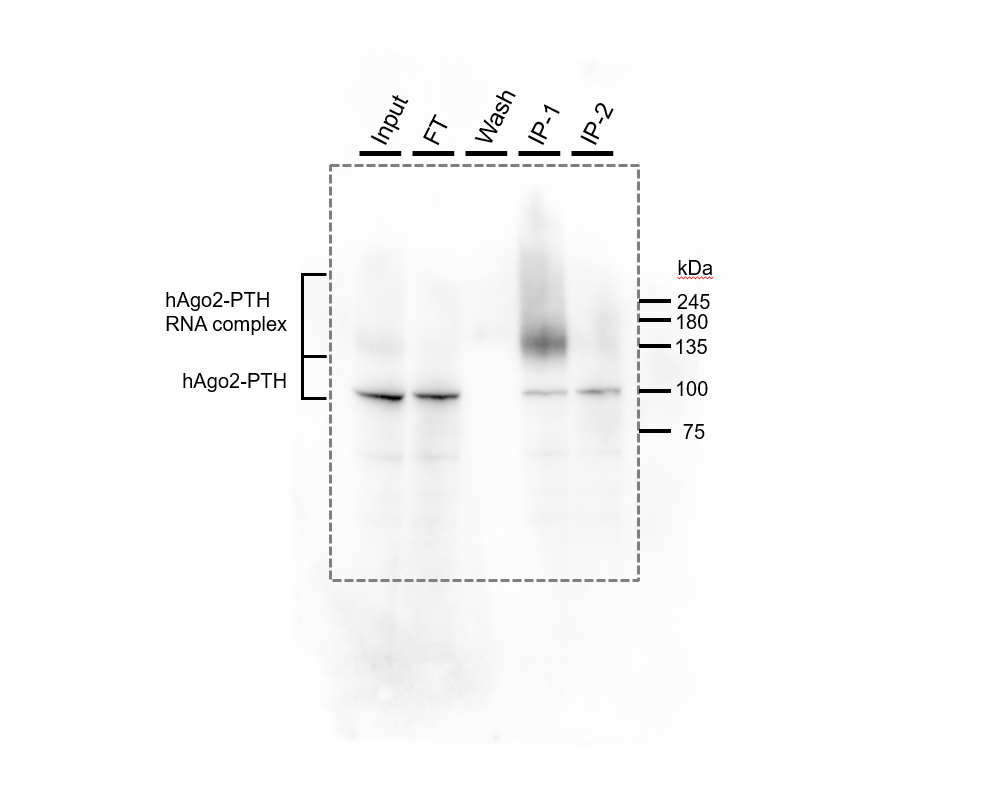


**Supplementary Figure S9:** Raw full gel image of *Supplementary Figure S2*

Western blot analysis of hAGO2-PTH precipitation from HEK293T cells after UV crosslinking. The migration position of AGO2-PTH was detected with AGO2-specific antibodies (Rat Monoclonal-clone 11A9, SAB4200085 Sigma-Aldrich) at concentration 1.5 μg/ml in PBS-T and incubated overnight at 4°C. As the secondary antibody we used Goat-Anti Rat IgG-HRP conjugate (sc-2006) at 1:5000 dilution in PBS-T for 1 hour at room temperature and developed with Pierce™ ECL Western Blotting Substrate (Catalog number: 32209). The chemiluminesce signal was detected by UVITEC Cambridge gel documentation system- Alliance 4.7 by using UVITEC-Alliance software with default settings of the lens parameters.

**Supplementary References**

1. Libri V, Helwak A, Miesen P, Santhakumar D, Borger JG, Kudla G, et al. Murine cytomegalovirus encodes a miR-27 inhibitor disguised as a target. Proceedings of the National Academy of Sciences. 2012;109:279–84.

2. Cristea IM, Williams R, Chait BT, Rout MP. Fluorescent proteins as proteomic probes. Mol Cell Proteomics. 2005;4:1933–41.

3. Smith T, Heger A, Sudbery I. UMI-tools: modeling sequencing errors in Unique Molecular Identifiers to improve quantification accuracy. Genome Res. 2017;27:491–9.

4. Andrews S. FASTQC. A quality control tool for high throughput sequence data. Available online at: http://www.bioinformatics.babraham.ac.uk/projects/fastqc/. 2010.

5. Davis MPA, van Dongen S, Abreu-Goodger C, Bartonicek N, Enright AJ. Kraken: a set of tools for quality control and analysis of high-throughput sequence data. Methods. 2013;63:41–9.

6. Bolger AM, Lohse M, Usadel B. Trimmomatic: a flexible trimmer for Illumina sequence data. Bioinformatics. 2014;30:2114–20.

7. Dobin A, Davis CA, Schlesinger F, Drenkow J, Zaleski C, Jha S, et al. STAR: ultrafast universal RNA-seq aligner. Bioinformatics. 2013;29:15–21.

8. Ramírez F, Dündar F, Diehl S, Grüning BA, Manke T. deepTools: a flexible platform for exploring deep-sequencing data. Nucleic Acids Res. 2014;42 Web Server issue:W187-191.

9. Lorenz R, Bernhart SH, Höner Zu Siederdissen C, Tafer H, Flamm C, Stadler PF, et al. ViennaRNA Package 2.0. Algorithms Mol Biol. 2011;6:26.

10. Wang L, Wang S, Li W. RSeQC: quality control of RNA-seq experiments. Bioinformatics. 2012;28:2184–5.

11. Broad Institute. Picard Toolkit. http://broadinstitute.github.io/picard/. 2018.

12. Okonechnikov K, Conesa A, García-Alcalde F. Qualimap 2: advanced multi-sample quality control for high-throughput sequencing data. Bioinformatics. 2016;32:292–4.

13. Chu J, Sadeghi S, Raymond A, Jackman SD, Nip KM, Mar R, et al. BioBloom tools: fast, accurate and memory-efficient host species sequence screening using bloom filters. Bioinformatics. 2014;30:3402–4.

14. Heng L. seqtk Toolkit for processing sequences in FASTA/Q formats. https://github.com/lh3/seqtk. 2012.

15. Liao Y, Smyth GK, Shi W. featureCounts: an efficient general purpose program for assigning sequence reads to genomic features. Bioinformatics. 2014;30:923–30.

16. Love MI, Huber W, Anders S. Moderated estimation of fold change and dispersion for RNA-seq data with DESeq2. Genome Biol. 2014;15:550.

17. Wickham H. ggplot2. Wiley Interdisciplinary Reviews: Computational Statistics. 2011;3:180–5.

18. Klimentová E, Hejret V, Krčmář J, Grešová K, Giassa I-C, Alexiou P. miRBind: A Deep Learning Method for miRNA Binding Classification. Genes (Basel). 2022;13:2323.
